# Psychometric Study of the Scale for the Assessment of Developmental Assets in the Neighborhood in a Sample of Chilean Adolescents

**DOI:** 10.3389/fpsyg.2020.00972

**Published:** 2020-06-12

**Authors:** Daniela Vera-Bachmann, José L. Gálvez-Nieto, Italo Trizano-Hermosilla, Sonia Salvo-Garrido, Karina Polanco

**Affiliations:** ^1^Department of Psychology, Austral University of Chile, Valdivia, Chile; ^2^Department of Social Work, University of La Frontera, Temuco, Chile; ^3^Department of Psychology, Temuco Catholic University, Temuco, Chile

**Keywords:** adolescence, neighborhood, positive development, validity, reliability

## Abstract

The Scale for the Assessment of Developmental Assets in the Neighborhood (SADAN) has shown acceptable psychometric properties for use in Spain and Chile. However, the original factor structure of five correlated factors and a second-order factor is not yet entirely clear. This study aimed to evaluate the scale’s psychometric properties of reliability and validity in a sample of Chilean adolescents. A cross-sectional design was used, with a sample of 2638 students (female = 49.1%) with an average age of 15.79 years (*SD* = 1.35). The results obtained when evaluating different confirmatory factor models show that the best structure is that of five correlated factors. We carry out a multigroup factor analysis up to the level of scalar invariance. We applied this analysis to the following groups: sex, type of school, and age. We conclude that the original version of the scale can be used in the Chilean context with slight modifications as it is necessary to deepen the validity evidence with external criteria.

## Introduction

An attractive frame of reference for understanding adolescence is the Positive Youth Development (PYD) approach (Benson et al., 2006; Oliva et al., 2008). Palomar-Lever and Victorio-Estrada (2014) understood adolescence as a stage of high plasticity, in which the relationships established with others and with the environment are fundamental aspects, for example, the neighborhood (Gálvez-Nieto et al., 2018).

Adolescents who have a positive perception of neighborhood security and social cohesion between neighbors show better psychological adjustment (Snedker and Herting, 2013; Mmari et al., 2014; Elliott et al., 2016; Vilhjalmsdottir et al., 2016). They also show higher life satisfaction (Maurizi et al., 2013; Rudolph et al., 2014; Rabinowitz et al., 2016), higher levels of prosocial behavior (Lenzi et al., 2013; Drinkard, 2014), a lower prevalence of problematic substance use (Tucker et al., 2013; Fuentes et al., 2015), and fewer disruptive behaviors (Chilenski, 2011).

There are psychometric tests to assess the perception that residents have of their neighborhood (Oliva et al., 2012a) even though most of these tests do not consider favorable aspects of adolescent development and adjustment (Herrero and Gracia, 2007). For this reason, the Scale for the Assessment of Developmental Assets in the Neighborhood (SADAN) provides an alternative to evaluate the perception of adolescents regarding different resources in the neighborhood that favor adolescent well-being.

Scale for the Assessment of Developmental Assets in the Neighborhood was developed from the positive youth development approach, and it assessed the social relationships established by adolescents in their neighborhood (Seligman and Csikszentmihalyi, 2000). It contributes to the psychological field as a measure for psychosocial evaluation in public health areas and promoting positive youth development.

Oliva et al. (2012a) developed and validated SADAN in Spain, analyzing the psychometric properties in a sample of 2400 adolescents from 17 educational centers. They perform an exploratory factor analysis using the Pearson matrix, the factorization method of the principal axis, and the oblimin rotation. They found a structure of five related factors that explained 62% of the variance. Subsequently, these authors carried out confirmatory factor analysis using the Pearson correlation matrix and the maximum likelihood estimation method. The evidence provided supported a second-order factor structure and five correlated factors. Oliva et al. (2012b) analyzed the relationships between some dimensions of SADAN and other factors, such as internalizing and externalizing problems, substance use, and life satisfaction. Subsequently, Gálvez-Nieto et al. (2019) carried out a study in Chile with a sample of 841 adolescents from 11 educational centers, which proposed a structure of five correlated factors. Considering that the structure is not clear in this population, the current study seeks to deepen the psychometric evidence in a larger sample.

Thus, the first hypothesis states that SADAN will maintain a second-order structure and five correlated factors. Furthermore, the scale scores will present adequate levels of reliability. The second hypothesis states that the scale will remain equivalent up to the level of scalar invariance for the variables sex, type of education, and age. The first objective of this study is to evaluate the psychometric properties of SADAN in a sample of Chilean adolescents. The second objective is to analyze the degree of invariance for the groups: sex, type of education, and age.

## Method

### Participants

We performed a stratified random sampling with a confidence interval of 95%, a variance of *p* = *q* = 0.50, and a margin of error of 3.30% (Scheaffer et al., 1987). As strata, we consider region, level of study (9th to 12th grade), and administrative dependency of the educational centers (public, private subsidized, and private). The sample (Table 1) corresponds to 2638 students of both sexes (50.9% male and 49.1% female) from 32 secondary schools, aged between 12 and 20 years (mean = 15.79 years, *SD* = 1.35). The students represent five zones in Chile: North (Antofagasta = four schools), North Central (Coquimbo = three schools), Central (Metropolitana = 16 schools), South Central (La Araucanía = seven schools), and South (Magallanes = two schools). The selected schools included students from various socioeconomic levels but mainly represented low and medium levels. Given the geographical characteristics of the country, we decided to exclude cities that contained less than 30% of the region’s population.

**TABLE 1:** Characteristics of the sample.

Region	Population	Population %	Sample
Region of Antofagasta	32,475	6.68%	188
Region of Coquimbo	39,26	8.07%	82
Metropolitan Region	351,791	72.3%	1,616
Region of the Araucanía	54,573	11.2%	684
Region of Magallanes and Chilean Antarctica	8,328	1.7%	68
**Total**	486,427	100%	2,638
**Type of teaching**			
Scientific-humanistic	337,009	69.3%	1,828
Technical-professional	149,418	30.7%	810
Total	486,427	100%	2,638
**Type of school**			
Public	142,276	29.25%	1,499
Private subsidized	292,134	60.06%	885
Private	52,017	10.69%	254
**Total**	486,427	100%	2,638

### Instruments

A sociodemographic questionnaire of multiple-choice questions was applied to document sex, age, date of birth, family origin (urban/rural), ethnic origin, grade level, and SADAN (Oliva et al., 2012a; Gálvez-Nieto et al., 2019).

Scale for the Assessment of Developmental Assets in the Neighborhood is a 22-item self-reporting instrument focused on the assessment of adolescents’ perception of their neighborhood. It uses an ordinal scale of seven points (1 = strong false, 7 = strong true). It is composed of five first-order factors: support and empowerment, attachment to the neighborhood, security, social control, and youth activities. It also uses a second-order factor that groups the five dimensions, called neighborhood assets.

### Procedure

To perform this study, we contacted the principals of the schools to request authorization to sample the students. To safeguard the ethical principles of the project, we gathered informed consent from students and parents or guardians. The research was approved and supported by the Scientific Ethics Committee at the Universidad de La Frontera. The instruments were applied voluntarily and anonymously during the first morning class.

### Data Analysis

We use confirmatory factor analysis (CFA) models to assess the factor structure of the scale. Following Asún et al. (2016), we used polychoric correlation matrix with the weighted least square with mean and variance adjusted (WLSMV) estimation method (Finney and Di Stefano, 2006). In this way, and considering the previous psychometric studies, in this study, we evaluate four models: one-dimensional, bifactor, second-order, and five correlated factors. We use the following several indices to analyze the goodness of the data fit by the different models. These indices are comparative fit index (CFI), Tucker–Lewis index (TLI), and root mean square error of approximation (RMSEA). In the case of the CFI and TLI indexes, values greater than or equal to 0.90 were considered a reasonable adjustment (Schumacher and Lomax, 1996), and values greater than 0.95 were considered a fine adjustment (Schreiber et al., 2006). For RMSEA, values less than 0.08 were considered a reasonable fit (Browne and Cudeck, 1993) and values less than 0.06 an excellent fit (Schreiber et al., 2006). Subsequently, we evaluate the degree of factorial invariance using the WLSMV estimation method for the groups (sex, type of school, and age). This analysis considers the following models (Vandenberg and Lance, 2000): M0 configural (equal number of factors), M1 metric (equal factor loadings), and M2 scalar (equality of intercepts). When we met the level of scalar invariance, we compared the latent means of the factors, setting those of the reference group to zero.

Finally, and based on the structure previously found, the reliability of each of the factors was determined using the following estimators: McDonald’s Omega and Cronbach’s alpha (Green and Yang, 2015; Trizano-Hermosilla and Alvarado, 2016). Additionally, following the recommendations of Domínguez-Lara and Merino-Soto (2015), the confidence intervals (CI) were estimated with the R 3.6 software for both Omega and Alpha using the “MBESS” (Kelly and Lai, 2019) and “Psych” (Revelle, 2020) packages, respectively.

## Results

### Descriptive Analysis

Table 2 presents the descriptive statistics of the items on the scale. When evaluating the means, we observed that item 13 obtained the highest score (*M* = 5.47, *SD* = 1.807). In contrast, item 22 presented the lowest mean (*M* = 2.86, *SD* = 1.816).

**TABLE 2:** Descriptive statistics.

Items	Mean	Std. Deviation	Skewness	Kurtosis
1. The adults in my neighborhood are concerned with the well-being of the youth/Las personas adultas de mi barrio se preocupan de que los jóvenes estemos bien	3.65	1.825	0.072	–1.032
2. People my age can find adults in my neighborhood to help solve a problem/La gente de mi edad puede encontrar en mi barrio personas adultas que le ayuden a resolver algún problema	3.97	1.763	–0.188	–0.949
3. The adults in my neighborhood say that young people must be heard/Las personas adultas de mi barrio dicen que hay que escuchar a los jóvenes	3.29	1.679	0.218	–0.854
4. I identify with my neighborhood/Me siento identificado con mi barrio	3.67	2.023	0.121	–1.229
5. Adults in my neighborhood value the youth/La gente adulta de mi barrio valora mucho a los jóvenes	3.51	1.715	0.124	–0.854
6. The adults in my neighborhood reprimand us if we damage trees or public gardens/Las personas adultas de mi barrio nos retan si estropeamos los árboles o jardines	4.36	1.968	–0.374	–1.011
7. I feel I am part of my neighborhood/Siento que formo parte de mi barrio	3.97	2.048	–0.096	–1.242
8. I feel very connected to my neighborhood/Me siento muy unido a mi barrio	3.56	2.023	0.172	–1.201
9. Living in my neighborhood makes me feel that I am part of a community/Vivir en mi barrio me hace sentir que formo parte de una comunidad	3.72	1.980	0.048	–1.189
10. In my neighborhood, when adults make decisions that affect young people, they listen to the youths’ opinions/En mi barrio, cuando las personas adultas toman decisiones que nos afectan a los jóvenes, escuchan antes nuestra opinión	3.14	1.761	0.345	–0.839
11. In my neighborhood, there are people who sell drugs/En mi barrio hay gente que vende droga	4.26	2.261	–0.115	–1.464
12. During vacation, there are many activities for young people to have fun with in my neighborhood/En vacaciones, en mi barrio hay muchas actividades para que podamos divertirnos los jóvenes	3.08	1.914	0.485	–0.982
13. Some of my friends are afraid to come to my neighborhood/Algunos amigos de fuera tienen miedo de venir a mi barrio	5.47	1.807	–0.942	–0.290
14. People in my neighborhood commit crimes and hooliganism/La gente de mi barrio comete delitos y ordinarieces (orinar en la calle, escupir, etc.)	4.62	2.126	–0.311	–1.305
15. The adults in my neighborhood would try to prevent young people from burning or breaking things (trashcan, etc.)/Las personas adultas de mi barrio tratarían de impedir que los jóvenes quemaran o rompieran cosas (papeleras, contenedores, etc.)	4.74	1.943	–0.587	–0.788
16. People of my age feel valued by adults in the neighborhood/La gente de mi edad nos sentimos apreciados por las personas adultas del barrio	3.60	1.777	0.090	–0.831
17. If a young person in my neighborhood tried to damage a car, an adult would try to stop him/her/Si un joven de mi barrio intentara dañar un auto las personas adultas lo evitarían	5.05	1.832	–0.806	–0.324
18. In my neighborhood, if you get into hooliganism, an adult will scold you/En mi barrio si haces cualquier ordinariez seguro que algún adulto te retará o llamará la atención	4.47	1.931	–0.400	–0.945
19. Young people in my neighborhood have places to get together during bad weather/19. Los jóvenes de mi barrio tenemos lugares donde reunirnos cuando hace mal tiempo.	3.15	1.958	0.431	–1.040
20. The young people in my neighborhood can do so many things after school that rarely get bored/Los jóvenes de mi barrio podemos hacer tantas cosas después de clase que raramente nos aburrimos	3.07	1.909	0.493	–0.904
21. In my neighborhood, there are often fights between street gangs/En mi barrio suele haber peleas entre bandas callejeras	5.25	1.940	–0.775	–0.674
22. There are few neighborhoods, such as my own, where there are as many activities for Young people/Hay pocos barrios en los que haya tantas actividades para jóvenes como en el mío	2.86	1.816	0.615	–0.660

### Factor Structure Evaluation

**FIGURE 1:**
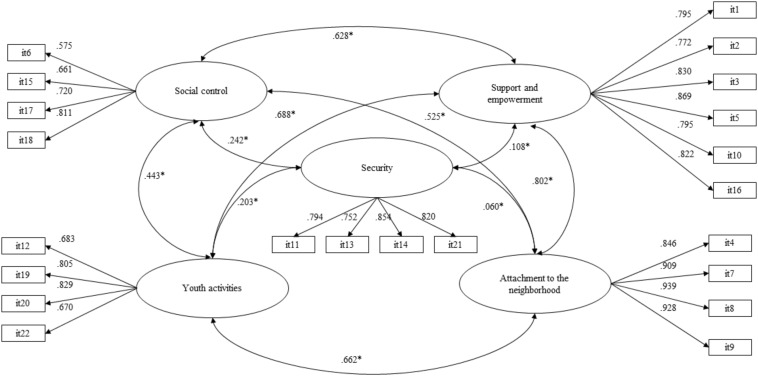
Confirmatory factor structure SADAN, *p < 0.001.

To evaluate the factor structure, four CFA models were estimated with the 22 items on the scale. The first model tested was the one-dimensional model, which provided an unsatisfactory fit [WLSMV – χ^2^ (*df* = 209) = 33,037.671; CFI = 0.668; TLI = 0.634 RMSEA = 0.244 (C.I = 0.242–0.249)]. The second one tested was a bifactor model, and the results again showed unsatisfactory indices [WLSMV – χ2 (*df* = 187) = 4194.737; CFI = 0.960; TLI = 0.950 RMSEA = 0.090 (C.I = 0.08–0.093)]. The third model evaluated a second order structure that grouped five factors, and the results for this model were unsatisfactory [WLSMV – χ^2^ (*df* = 204) = 4902.601; CFI = 0.953; TLI = 0.946 RMSEA = 0.093 (C.I = 0.091–0.096)]. Finally, the estimation of a model of five correlated factors revealed an acceptable fit [WLSMV – χ^2^ (*df* = 199) = 3499.280; CFI = 0.967; TLI = 0.961 RMSEA = 0.079 (C.I = 0.077–0.082)]. From these results, it is possible to point out that the model that best fits the data is that of five correlated factors (Figure 1). As can be observed, all the factors of the scale present significant and positive correlations with values that range between a minimum of 0.060 for security and attachment to the neighborhood and a maximum of 0.802 between the dimensions of attachment to the neighborhood and support and empowerment.

### Factorial Invariance

Once the factorial structure of the scale was confirmed, we carried out factor invariance analysis for the groups sex, type of education, and age. As shown in Table 3, we evaluate the configuration invariance or M0, resulting in all satisfactory goodness-of-fit indices and statistically significant parameters. This result corroborated the structure of five correlated factors and the same items for the variables sex, type of education, and age.

**TABLE 3:** Factorial invariance.

Variable	Model	WLSMV-χ^2^ (df)	CFI	TLI	RMSEA	Comp.	ΔWLSMV-χ^2^	Δ*df*	*p*-value (ΔWLSMV-χ^2^)	ΔCFI
Sex	1 M0	3663.237 (398)*	0.966	0.961	0.079					
	2 M1	3649.266 (415)*	0.967	0.963	0.077	*2* vs. *1*	31.691	17	0.0164	–0.001
	3 M2	3616.944 (520)*	0.969	0.972	0.067	*3* vs. *2*	179.651	105	< 0.001	–0.002
Type of education	1 M0	3652.427 (398)*	0.967	0.962	0.079					
	2 M1	3636.356 (415)*	0.967	0.964	0.077	*2* vs. *1*	27.662	17	0.0496	0
	3 M2	3609.877 (520)*	0.969	0.972	0.067	*3* vs. *2*	183.246	105	< 0.001	–0.002
Age	1 M0	3636.880 (398)*	0.967	0.962	0.079					
	2 M1	3617.988 (415)*	0.967	0.964	0.076	*2* vs. *1*	29.773	17	0.0280	0
	3 M2	3591.873 (520)*	0.969	0.972	0.067	*3* vs. *2*	180.326	105	< 0.001	–0.002

*WLSMV-χ^2^, Chi-Square obtained with the WLSMV method; *df*, degrees of freedom; *TLI*, Tucker-Lewis fit index; *CFI*, comparative adjustment index; *RMSEA*, root mean square error of approximation; ΔWLSMV-χ^2^, change in Chi-Square obtained with the WLSMV method; Δ*df*, change in degrees of freedom; Δ*CFI*, Change in CFI; ^∗^*p* < 0.001.*

Subsequently, the M1 metric invariance model was evaluated, adding restrictions to the factor loadings. The results indicate that there are no statistically significant differences between the configuration and metric models for the variables sex (*p*-ΔWLSMV - χ^2^ = 0.0164; ΔCFI = −0.001), type of education (*p*-ΔWLSMV - χ^2^ = 0.0496; ΔCFI = 0), and age (*p*-ΔWLSMV - χ^2^ = 0.0280; ΔCFI = 0).

Next, the level of M2 scalar invariance was analyzed, including constraints on the item intercepts to be equivalent in the groups. The results indicated that there are statistically significant differences between the metric and scalar invariance models for the variable sex (*p*-ΔWLSMV - χ^2^ < 0.001; ΔCFI = −0.002), type of education (*p*-ΔWLSMV - χ^2^ < 0.001; ΔCFI = −0.002), and age (*p*-ΔWLSMV - χ^2^ < 0.001; ΔCFI = −0.002). However, with the CFI difference criterion, the equality between both models is accepted.

Also, we evaluated the level of latent means between these groups. Results show statistically significant differences. In particular, females present the lowest levels for support and empowerment (−0.139, *p* < 0.001), attachment to the neighborhood (−0.219, *p* < 0.001), security (−0.102, *p* = 0.004), and youth activities (−0.208, *p* < 0.001). We did not observe statistical differences in the social control factor (*p* = 0.229).

For the type of education group, we also observed statistically significant differences in favor of scientific-humanistic schools in comparison to technical-professional schools for the factors of support and empowerment (−0.088, *p* = 0.008), security (−0.474, *p* < 0.001), and social control (−0.139, *p* < 0.001). There were no statistically significant differences in the factors of attachment to the neighborhood (*p* = 0.064) and youth activities (*p* = 0.146).

Finally, when comparing the latent means for the age group, statistically significant differences were observed in favor of students located in the age range between 12 and 15 years. Students aged between 16 and 19 years show the lowest levels in the latent dimensions of support and empowerment (−0.137, *p* < 0.001), attachment to the neighborhood (−0.132, *p* < 0.001), security (−0.220, *p* < 0.001), social control (−0.079, *p* = 0.001), and youth activities (−0.064, *p* = 0.038).

### Evidence of Reliability

For the latent factor, attachment to the neighborhood, reliability values were obtained with the omega coefficient of 0.932 (C.I = 0.926−0.938) and Cronbach’s alpha 0.931 (C.I = 0.927−0.935). For support and empowerment, we observed an omega coefficient of 0.903 (C.I = 0.895−0.909) and an alpha of 0.902 (C.I = 0.897−0.907). For security, we obtained an omega value of 0.840 (C.I = 0.828−0.851) and an alpha of 0.836 (C.I = 0.827−0.845). For the youth activities, the omega coefficient obtained was 0.786 (C.I = 0.770−0.801), and the alpha value was 0.784 (C.I = 0.772−0.796). Finally, for social control, the omega value was 0.736 (C.I = 0.716−0.755), and the alpha was 0.734 (C.I = 0.719−0.748). Therefore, we observed similar values for both reliability estimators as well as in their confidence intervals. Additionally, the tests showed adequate values of reliability for the five dimensions of the questionnaire.

## Discussion

This research had two objectives. The first objective was to evaluate the psychometric properties of SADAN in a sample of Chilean adolescents. The second was to analyze the degree of invariance for sex, type of education, and age. The results obtained show partial compliance with the first hypothesis, which stated that the scale scores would maintain a second-order structure and five correlated factors in addition to adequate levels of reliability. The second hypothesis had complete compliance and stated that the scale scores would be equivalent up to the level of scale invariance for the variables sex, type of education, and age.

The results of this study do not support the first hypothesis in the sense that the scale would maintain the second-order factor structure and five correlated factors. Instead, these results confirmed the presence of a structure of five correlated factors for the Chilean sample (Gálvez-Nieto et al., 2019).

It is interesting to note that, when the results of the original study were analyzed (Oliva et al., 2012a), it was observed that the second-order structure obtained a low loading factor for security (0.29), considering the factor loadings of the rest of the dimensions, support and empowerment (0.84), attachment to the neighborhood (0.70), social control (0.46), and youth activities (0.53), which are comparatively higher. The mismatch of the second-order model could be due to the questionnaire items being phrased negatively. Hence, we suggest that future psychometric studies modify the question phrasing.

The results of the invariance models show that SADAN applies to both males and females. However, latent trait levels differ significantly in the means of the following factors: support and empowerment, attachment to the neighborhood, security, and youth activities. In this study, we found that the latent means of these factors are higher in males, which is consistent with the findings obtained by the study by Gálvez-Nieto et al. (2019). These differences could be explained because adolescent females use and appropriate public space differently than males. In practical terms, females prefer safer spaces, showing similar behavior to adults supervising children and older adults (Pérez, 2012). Females also tend to perceive crimes of different kinds more frequently. They also have less control over threatening situations as well as a more significant number of experiences of victimization, both direct and indirect, increasing their perception of insecurity (Espinoza and Monge-Nájera, 2017; Valera-Pertegas and Guardia-Olmos, 2017).

The results of the factor invariance analysis in scientific-humanistic and technical-professional schools report that the SADAN applies for both school contexts. However, the latent means of the factors support and empowerment, security, and social control differ significantly, obtaining higher average scores from the students in scientific-humanistic schools. These differences can be explained because students who choose technical-professional schools present significantly lower socioeconomic levels. This condition is associated with neighborhoods with more critical problems of security, support and empowerment, and social control (Bellei et al., 2017).

The factor invariance analysis applied according to the group age of the students showed equivalence up to the level of scalar invariance. It shows significant differences in the levels of the latent means of the five factors of the instrument, obtaining higher averages for the students belonging to the younger age group. These results coincide with studies that evaluate psychosocial variables among students of different ages, who state that younger students tend to report a better assessment of the characteristics of their neighborhood (Gálvez-Nieto et al., 2020).

Regarding the limitations of the present study, we suggest continuing to expand the line of research to include other measures to assess the convergent and divergent validity of SADAN. On the other hand, the results of this study should be interpreted with caution because only a self-reporting instrument was used as an indicator. We feel that it would be interesting to evaluate not only the perception of the students but also the perspective of the parents or guardians of the adolescents. Another limitation to consider when interpreting the results is the overrepresentation of the public schools, which participated in a higher number than the other types of educational schools.

Future lines of research should analyze the invariance of the construct in other Latin American student populations.

## Data Availability Statement

The datasets generated for this study are available on request to the corresponding author.

## Ethics Statement

The studies involving human participants were reviewed and approved by the Comité de Ética de la Universidad de La Frontera. Written informed consent to participate in this study was provided by the participants’ legal guardian/next of kin.

## Author Contributions

DV-B conducted bibliographic search, theoretical framework, integrated results, and contributed to the discussion. JG-N performed the data collection, methodological design, contributed to analysis, results, and discussion. IT-H contributed to the methodological design, performed the data analysis, and generated the results. SS-G review of the analysis of the invariance models and general review of the manuscript. KP conducted bibliographic search, theoretical framework, and contributed to the discussion.

## Conflict of Interest

The authors declare that the research was conducted in the absence of any commercial or financial relationships that could be construed as a potential conflict of interest.
